# Exosomes from miR-374a-5p-modified mesenchymal stem cells inhibit the progression of renal fibrosis by regulating MAPK6/MK5/YAP axis

**DOI:** 10.1080/21655979.2022.2033465

**Published:** 2022-02-09

**Authors:** Mingzhu Liang, Di Zhang, Danna Zheng, Wenfang He, Juan Jin

**Affiliations:** a Department of Nephrology, The Medical College of Qingdao University, Qingdao, Shandong, China; b Nephrology Center, Department of Nephrology, Zhejiang Provincial People’s Hospital and Affiliated People’s Hospital, Hangzhou Medical College, Hangzhou, Zhejiang, China

**Keywords:** Renal fibrosis, mesenchymal stem cells (MSC), exosomes, miR-374a-5p, MAPK6

## Abstract

Chronic kidney disease (CKD) in clinical is defined as a gradual loss of kidney function for more than 3 months. The pathologic course of CKD is characterized by extensive renal fibrosis; thus, preventing renal fibrosis is vital for the treatment of CKD. It has been reported that microRNA (miR)-374a-5p was under-expressed in renal venous blood samples from patients with CKD. In addition, it exhibited anti-apoptotic effects in renal tissues suggesting that miR-374a-5p may play an important role in CKD. However, it is not clear whether miR-374a-5p could be delivered to renal cells by exosomes and exerts anti-renal fibrosis effects. To mimic renal fibrosis *in vitro*, human renal tubular epithelial cell lines (HK-2 cells) were treated by transforming growth factor-β (TGF-β) 1. Reverse transcription-quantitative polymerase-chain reaction (RT-qPCR) or Western blot was carried out to evaluate the mechanism by which miR-374a-5p regulated the development of renal fibrosis. Next, exosomes were isolated using with ultracentrifugation method, and the relationship between miR-374a-5p and MAPK6 was evaluated using dual-Luciferase a reporter assay system. The results indicated TGF-β1 significantly down-regulated the expression of miR-374a-5p in HK-2 cells and miR-374a-5p agomir remarkably inhibited the progression of fibrosis *in vitro*. In addition, exosomal miR-374a-5p could be internalized by HK-2 cells and obviously enhanced the level of miR-374a-5p in HK-2 cells. Furthermore, exosomal miR-374a-5p prevented the progression of renal fibrosis *in vivo* by regulating MAPK6/MK5/YAP axis. In conclusion, exosomal miR-374a-5p inhibited the progression of renal fibrosis by regulating MAPK6/MK5/YAP axis.

## Introduction

Chronic kidney disease (CKD) in clinical is defined as a gradual loss of kidney function for more than 3 months [1–3]. The pathologic progression of CKD is characterized by extensive renal fibrosis due to the accumulation of extracellular matrix (ECM) [4–6]. Thus, early prevention of renal fibrosis is vital for the treatment of CKD [4]. Renal fibrosis is the process of renal fibrous tissue hyperplasia due to drug poisoning, hypertension, diabetes, inflammatory stimulation, cytokines and other pathogenic factors, and finally resulting in the loss of renal function [7–9]. In addition, renal fibrosis is characterized by enhanced reflection of the kidney, unclear boundary between cortex and medulla, and even renal shrinkage [7,9,10]. Although there have been a lot of scientific studies on renal fibrosis, the specific occurrence and development mechanism of renal fibrosis remain unclear.

MicroRNAs (miRNAs) are small and non-coding RNAs [11,12]. MiRNAs play a crucial role in many diseases including cancer, liver fibrosis, renal fibrosis, etc. [13–15]. Exosomes are small vesicles mainly composed of proteins and nucleic acids with a diameter of 30–150 nm [16, 17]. As intercellular communication carriers, exosomes could mediate the transport of biological macromolecules such as nucleic acids between cells, and extensively affect the body’s pathophysiological process [18,19]. For example, bone mesenchymal stem cells-derived exosome (BMSCs-exo) could inhibit lipopolysaccharide (LPS)-induced acute uterine injury (AUI) of endothelial progenitor cells [20]. Besides, exosomal miRNA-150-5p derived from BMSCs prevents cerebral ischemia/reperfusion (I/R) injury [21]. Additionally, exosomes play an indispensable role in the pathophysiological process of renal fibrosis [16,22,23]. For instant, exosomes can transfer miR-let7c from MSCs to NRK52E cells, thereby inhibiting the process of renal fibrosis [22]. Meanwhile, exosomal miR-29 was able to alleviate renal fibrosis [23].

It has been reported that microRNA (miR)-374a-5p was under-expressed in renal venous blood samples from patients with CKD. In addition, it exhibited anti-apoptotic effects in renal tissues suggesting that miR-374a-5p may play an important role in CKD [24]. Therefore, we aimed to explore whether miR-374a-5p could be delivered to renal cells by exosomes, and exerts anti-renal fibrosis effects in the current study. The results indicated that exosomal miR-374a-5p prevented the progression of renal fibrosis by regulating MAPK6/MK5/YAP axis for the first time. Therefore, the present study might provide a new therapeutic strategy for the treatment of renal fibrosis.

## Material and methods

### Cell culture

HK-2 cells were provided by American Type Culture Collection (ATCC, Manassas, VA, USA). Bone marrow MSCs were provided by iCell Bioscience Inc. These cells were maintained in DMEM (Thermo Fisher Scientific, Waltham, MA, USA) with 10% fatal bovine seru (FBS), 1% penicillin and 1% streptomycin in a 5% CO_2_ atmosphere at 37°C. To mimic renal fibrosis *in vitro*, HK-2 cells were dealt with 5 ng/mL TGF-β1 for 48 h [25].

### RT-qPCR

Trizol reagent (ELK Biotechnology, Wuhan, China) was carried out to measure total RNA in HK-2 cells and exosomes. EntiLink™ 1st Strand cDNA Synthesis Kit was carried out to synthesize cDNA. Then, a StepOne™ Real-Time PCR System was carried out to conduct qPCR. To analyze the expression of miR-374a-5p, the 2^−ΔΔCt^ method was carried out [26]. Primer sequences are as Table 1. U6 or β-actin was worked as internal controls for miR-374a-5p or MAPK6, respectively.

**Table 1.** Primer sequences

Name		Primer sequences (5’-3’)
miR-374a-5p	Forward	CCCGGGTTATAATACAACCTG
	Reverse	CTCAACTGGTGTCGTGGAGTC
MAPK6	Forward	CGTCAGGAGCTTCTCAGCGT
	Reverse	GGCTTGAAATTGGCTCATCC
U6	Forward	CTCGCTTCGGCAGCACAT
	Reverse	AACGCTTCACGAATTTGCGT
β-actin	Forward	GTCCACCGCAAATGCTTCTA
	Reverse	TGCTGTCACCTTCACCGTTC

### Western blot assay

Radio-Immunoprecipitation Assay (RIPA) buffer (Aspen Biotechnology, Wuhan, China) was carried out to extract total proteins from cells or from tissues. (Bicinchonininc acid) BCA kit (Aspen) was carried out to quantify the concentration of proteins. Then, the samples were loaded in 10% sodium dodecyl sulfate-polyacrylamide gel electrophoresis (SDS-PAGE) and followed by transferred to Polyvinylidene-Fluoride (PVDF) membrane. Next, the proteins were incubated with primary antibodies and then incubated with the horseradish peroxidase (HRP)-labeled secondary antibody. Finally, an efficient chemiluminescenc (ECL) kit was used to evaluate these samples [27]. In the currently study, these primary antibodies (Abcam, Cambridge, MA, USA) used were listed as follows: Collagen 1α1, Fibronectin, α-SMA, CD63, TSG101, p-MAPK6, MAPK6, p-MK5, MK5 and YAP. GAPDH was worked as internal controls [27].

### Exosomes isolation

The ultracentrifugation method was carried out to extract the exosomes from MSCs. MSCs supernatant was collected and centrifuged [28,29]. Then, the exosomes were obtained.

### Transmission electron microscopy (TEM) analysis

TEM was conducted to evaluate the number and morphology of collected vesicles. First, drop the exosomes sample on the carbon supporting membrane copper net for 5 min. Next, 2% phosphotungstic acid was dropped onto the carbon supported membrane copper net for 3 min. Finally, a TEM was used to observe the number and morphology of collected vesicles [29].

### Nanoparticle Tracking Analysis (NTA)

NTA analysis was comducted to confirm the particle size of collected vesicles. Firstly, the exosomes sample was cleaned using deionized water. Next, ZetaView analyzer (Particle Metrix, Meerbusch, Germany) was calibrated. After that, the collected vesicles were washed with PBS buffer twice. Finally, ZetaView analyzer was carried out to evaluate the particle size of collected vesicles [30,31].

### Flow cytometry assay

Annexin-V-FITC apoptosis detection kit was provided by Tianjin Sanjian Biotechnology Co., Ltd. (Tianjin, China). HK-2 cells were maintained in 6-well plates (5 × 10^4^/mL). After that, HK-2 cells were treated by FITC‐Annexin V for 15 min. Then, cells were treated with 5 μL propidium iodide (PI) for another 15 min in the darkness. Then, the apoptosis of HK-2 was evaluated using a flow cytometry [26].

### Dual-luciferase reporter assay

Either wild‐type (WT) or mutant (MT) MAPK6‐3′ Untranslated Regions (UTR) fragment was put into the pGL6-miRNA luciferase reporter vector (Beyotime, Shanghai, China). Next, MAPK6 (WT or MT) was transfected into HK-2 cells together with miR-374a-5p agomir or negative control (NC). Subsequently, dual-Luciferase a reporter assay system was carried out to confirm the relationship between miR-374a-5p and MAPK6 [32].

### Animal study

C57BL/6 mice weighing 18 ± 2 g were provided from Vital River (Beijing, China). This study was complies with the National Institutes of Health Guide (NIHG) for the Care and Use of laboratory animals. The mice were grouped as follows: Sham, Unilateral Uretera Obstruction (UUO), UUO + exosomes derived from MSCs (MSCs/NC-Exo) and UUO + exosomes derived from miR-374a-5p-modified MSCs (MSC/miR-374a-5p-Exo). To establish the model of UUO, mice were subjected to peritoneal injection anesthesia with pentobarbital sodium (1%). Then, mice were ligated at two points of the left ureter, and partially ligated to the ureteral-pelvic junction based on the available literature [33]. The sham group was used as a control. Twelve weeks later, mice were injected intravenously with MSCs/NC-Exo or MSC/miR-374a-5p-Exo twice weekly for 4 weeks [34]. At the end of study, mice were euthanasia via 40% volume/min CO_2_ and kidney tissue was collected from the mice.

### Hematoxylin-eosin (HE) staining

The kidney tissue was fixed with paraformaldehyde and embedded in paraffin. Then, the tissue was dewaxed with xylene and followed by dehydrated with 70%, 80% and 90% alcohol. After that, the tissue was stained using HE. Subsequently, the tissue was dehydrated with alcohol. Finally, the staining result was observed under an optical microscope [35].

### Measurement of blood urea nitrogen (BUN) and creatinine (CR)

The levels of BUN or CR were evaluated by Urea Assay Kit or Creatinine Assay kit provided by Jiancheng Bioengineering Institute respectively according to the manufacturer’s instructions [35,36].

### Immunohistochemistry (IHC) staining

Mice kidney tissues were fixed with paraformaldehyde. Then, the tissues were treated with 0.01 M boiling water citric acid buffer to extract antigens. Next, the tissues were treated with fluorescent labeled primary antibody (α-SMA). Subsequently, a fluorescence microscope was used to visualize the IHC staining, according to previous literature [37].

### Statistical analysis

Statistical data were analyzed by GraphPad Prism (La Jolla, CA, USA). All data were presented as mean ± standard deviation (SD). Differences in multiple groups were analyzed by one-way analysis of variance (ANOVA) and Tukey’s test [25,35].

## Results

### *MiR-374a-5p agomir remarkably inhibits TGF-β1-induced fibrosis* in vitro

**Figure 1.**
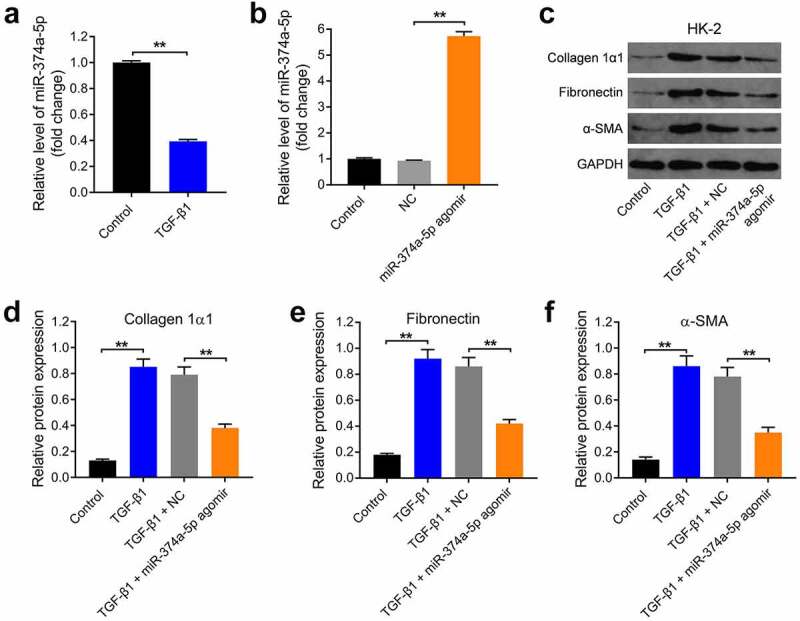
**MiR-374a-5p agomir remarkably inhibits TGF-β1-induced fibrosis *in vitro***. (a) HK-2 cells were treated with 5 ng/mL TGF-β1 for 48 h. RT-qPCR was used to evaluate the expression of miR-374a-5p in HK-2 cells. (b) HK-2 were dealed with miR-374a-5p agomir or NC using Lipofectamine® 2000 and the level of miR-374a-5p was evaluated with RT-qPCR. **(C, D, E and F)** Western blot were carried out to evaluate the levels of Collagen 1α1, Fibronectin and α-SMAs. **P < 0.01.

In order to mimic renal fibrosis *in vitro*, HK-2 cells were treated with TGF-β1. As shown in (Figure 1a), the level of miR-374a-5p was significantly downregulated by TGF-β1 in HK-2 cells and miR-374a-5p agomir obviously increased the level of miR-374a-5p in HK-2 cells (Figure 1b). In addition, TGF-β1 significantly increased the levels of Collagen 1α1, Fibronectin and α-SMA in HK-2 cells; however, these phenomena were significantly reversed by miR-374a-5p agomir (Figures 1c-f). Taken together, TGF-β1 downregulated the level of miR-374a-5p in HK-2 cells and miR-374a-5p agomir remarkably inhibits the TGF-β1-induced fibrosis *in vitro*.

### Exosomes from miR-374a-5p-modified MSC can be internalized by HK-2 cells

**Figure 2.**
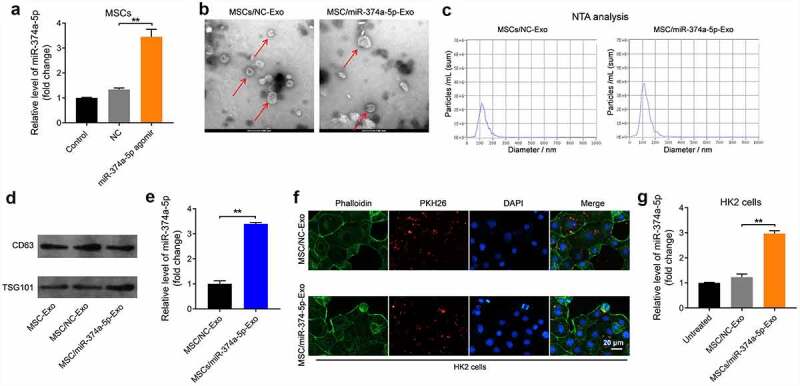
**Exosomes from miR-374a-5p-modified MSC can be internalized by HK-2 cells**. (a) MSCs were transfected with miR-374a-5p agomir NC or miR-374a-5p agomir using Lipofectamine® 2000 and RT-qPCR was conducted to evaluate the level of miR-374a-5p in MSCs. (b, c) TEM and NTA analysis were used to characterize the morphology and particle size of vesicles. (d) Western blot was used to evaluate the levels of CD63 and TSG101. (e) The level of miR-374a-5p in MSC/miR-374a-5p-Exo or MSCs/NC-Exo was evaluated by RT-qPCR. (f) HK-2 cells were incubated with PKH26-labeled MSC/miR-374a-5p-Exo or PKH26-labeled MSCs/NC-Exo with for 24 h. Then, a fluorescence microscope was conducted to observe PKH26 staining. Green color: HK-2 cells, Red color: exosome, blue color: nucleus. (g) HK-2 cells were treated with MSC/miR-374a-5p-Exo or MSCs/NC-Exo and the level of miR-374a-5p in HK-2 cells was evaluated by RT-qPCR. **P < 0.01.

It has been reported that exosomes from MSCs could inhibit the progression of renal fibrosis [38,39]. In order to explore if exosomal miR-374a-5p could better prevent the renal fibrosis, MSCs were transfected with miR-374a-5p firstly. The result of RT-qPCR suggested miR-374a-5p agomir markedly upregulated the level of miR-374a-5p in MSCs (Figure 2a). Then, exosomes were collected and characterized using with TEM and NTA. The data indicated these extracellular vesicles were discoid vesicles with a phospholipid bilayer structure and are 100 to 150 nm in diameter (Figures 2b,c); meanwhile, the isolated extracellular vesicles expressed specific exosome markers CD63 and TSG101 (Figure 2d). Moreover, the level of miR-374a-5p in exosomes derived from miR-374a-5p-modified MSCs was much higher than that in MSCs/NC-Exo (Figure 2e).

Next, to investigate whether these exosomes could be internalized by HK-2 cells, a cell membrane staining dye PKH26 was used. The data of staining indicated that both MSC/miR-374a-5p-Exo and MSCs/NC-Exo could be absorbed by HK-2 cells (figure 2f). After absorbing, MSC/miR-374a-5p-Exo obviously increased the expression of miR-374a-5p in HK-2 cells (Figure 2g). All in all, MSC/miR-374a-5p-Exo could be internalized by HK-2 cells and obviously upregulated the level of miR-374a-5p in cells.

### MSC/miR-374a-5p-Exo markedly inhibits the progression of fibrosis in vitro

**Figure 3.**
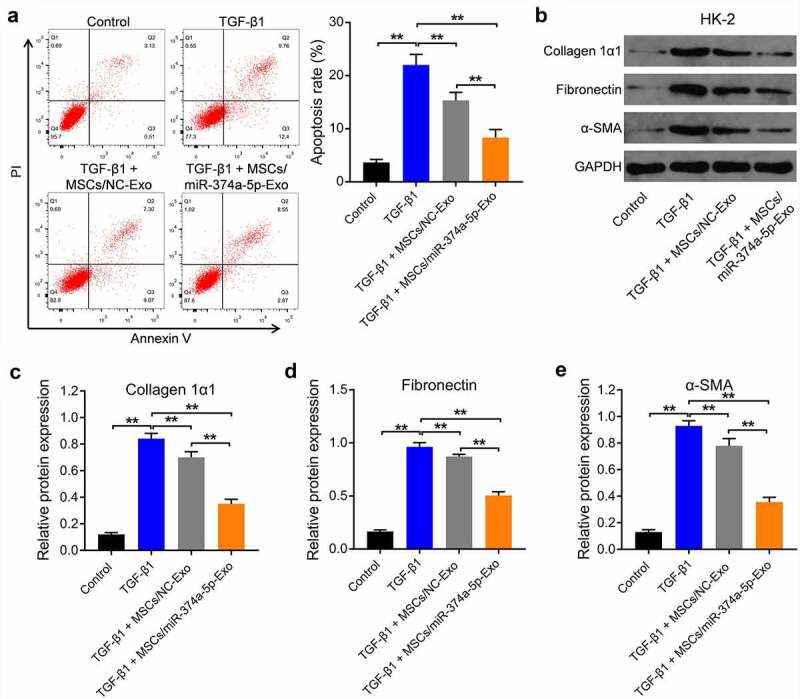
**MSC/miR-374a-5p-Exo markedly inhibits the progression of fibrosis *in vitro***. (a) Flow cytometry was carried out to measure the apoptosis. (b, c, d and e) Western blot assay were carried out to evaluate the levels of Collagen 1α1, Fibronectin and α-SMA in HK-2 cells. (Supplementary Figure 1A) Flow cytometry was carried out to measure the apoptosis. (Supplementary Figure 1B) Western blot assay were carried out to evaluate the levels of Collagen 1α1 and Fibronectin in HK-2 cells. **P < 0.01.

With the aim of investigating the effect of MSC/miR-374a-5p-Exo on the apoptosis of HK-2 cells, flow cytometry was carried out. As indicated in (Figure 3a), TGF-β1 clearly induced the apoptosis of HK-2 cells, and this phenomenon was reversed by MSC/miR-374a-5p-Exo. Meanwhile, TGF-β1 upregulated the levels of Collagen 1α1, Fibronectin and α-SMA in HK-2 cells, while these upregulations were partly reversed by MSC/miR-374a-5p-Exo (Figures 3b-e).

In order to verify the effect of miR-374a-5p on the progression of fibrosis, miR-374a-5p inhibitor was used. As suggested in supplementary Figure 1A, the effect of MSC/miR-374a-5p-Exo on the apoptosis of HK-2 cells was abolished by miR-374a-5p inhibitor. Expectantly, the effect of MSC/miR-374a-5p-Exo on the expressions of Collagen 1α1 and Fibronectin in HK-2 cells was abolished when miR-374a-5p was eliminated (Supplementary Figure 1B). All these results suggested that MSC/miR-374a-5p-Exo was able to inhibit TGF-β1-induced apoptosis of HK-2 cells and prevent the progression of fibrosis *in vitro*.

### MiR-374a-5p regulates MAPK6/MK5/YAP axis

**Figure 4.**
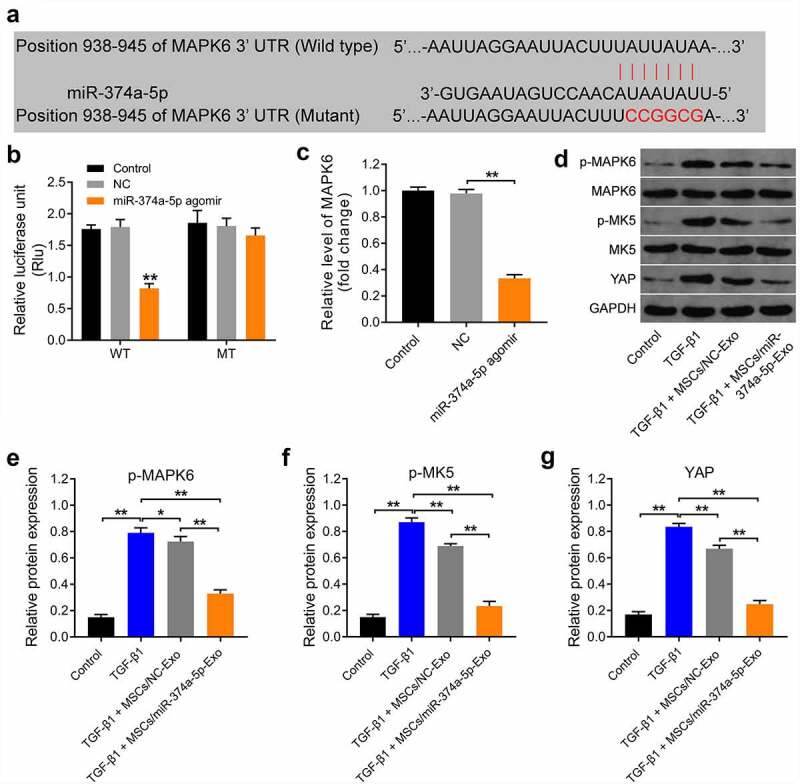
**MiR-374a-5p regulates MAPK6/MK5/YAP axis**. (a) MiRDB and TargetScan databases were used to predict the downstream targets of miR-374a-5p. (b) The relationship between miR-374a-5p and MAPK6 was explored by dual-luciferase reporter assay. (c) RT-qPCR was used to evaluate the level of MAPK6p in HK-2 cells. (d, e, f and g) Western blot assay were carried out to measure the expressions of p-MAPK6, MAPK6, p-MK5, MK5 and YAP in HK-2 cells. **P < 0.01.

To study the mechanism by which MSC/miR-374a-5p-Exo mediated the development of renal fibrosis, miRDB (http://www.mirdb.org/cgi-bin/search.cgi) and TargetScan (http://www.targetscan.org/vert_72/) online databases were used. These two databases commonly predicted that MAPK6 was the downstream targets of miR-374a-5p (Figure 4a); in addition, it has been reported that MAPK6 have a close relationship with kidney-related diseases [40,41]. Thus, we focused on investigating the relationship between miR-374a-5p and MAPK6 in the current study.

In addition, miR-374a-5p agomir notably downregulated the luciferase activity of cell harboring WT of MAPK6; however, it had no effect on the luciferase activity of cell transfected with mutant of MAPK6 (Figure 4b). Consistently, miR-374a-5p agomir markedly inhibited the gene expression of MAPK6 in HK-2 cells (Figure 4c). Moreover, TGF-β1 obviously increased the levels of p-MAPK6, p-MK5 and YAP in HK-2 cells, and these increases were all reversed by MSC/miR-374a-5p-Exo (Figures 4d-g). Taken together, miR-374a-5p regulate MAPK6/MK5/YAP axis by directly binding with mRNA of MAPK6 in HK-2 cells.

### MSC/miR-374a-5p-Exo inhibits the progression of renal fibrosis in vivo

**Figure 5.**
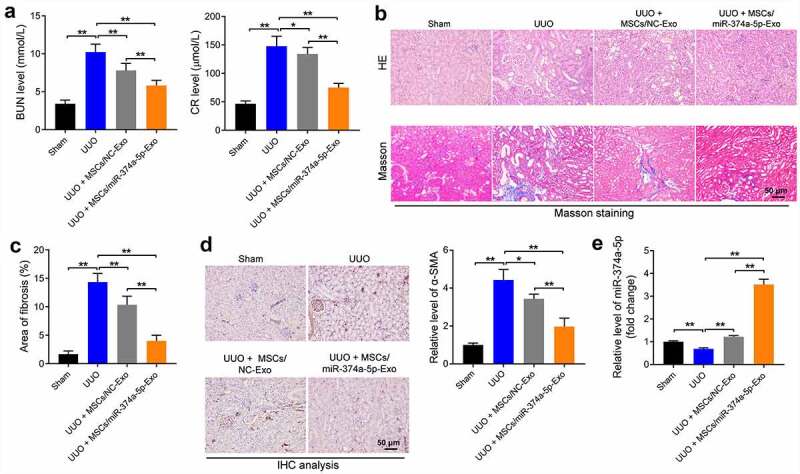
**MSC/miR-374a-5p-Exo prevents the progression of renal fibrosis**. (a) The level of BUN or CR was evaluated by urea assay kit or creatinine assay kit. (b, c) The tissue structure, cell morphology, inflammatory infiltration and fibrosis of kidney tissue were observed by HE and Masson staining. (d) The level of α-SMA in UUO mouse renal tissue was evaluated by IHC staining. (e) RT-qPCR was conducted to evaluate the expression of miR-374a-5p in mouse renal tissue. **P < 0.01.

Next, to further investigate the effect of MSC/miR-374a-5p-Exo on the process of renal fibrosis *in vivo*, UUO mouse model was established. As indicated in Figure 5a, the levels of BUN and CR were notably increased in UUO mice, while MSC/miR-374a-5p-Exo treatment brought them back to the normal situation (Figure 5a). In addition, the areas of inflammatory infiltration and fibrosis were upregulated in the UUO group, whereas, MSC/miR-374a-5p-Exo alleviated these lesion (Figures 5b,c). Consistently, the level of α-SMA was upregulated in UUO mouse renal tissue, while this upregulation was visibly reversed by MSC/miR-374a-5p-Exo (Figure 5d). Moreover, MSC/miR-374a-5p-Exo significantly promoted the expression of miR-374a-5p in UUO mouse renal tissue (Figure 5e). To sum up, MSC/miR-374a-5p-Exo significantly prevented the development of renal fibrosis.

### MSC/miR-374a-5p-Exo blocks the progression of renal fibrosis by regulating MAPK6/MK5/YAP axis *in vivo*

**Figure 6.**
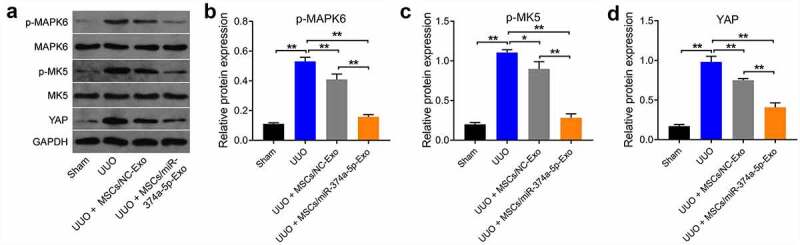
**MSC/miR-374a-5p-Exo blocks the renal fibrosis *in vivo* by regulating MAPK6/MK5/YAP axis**. (a, b, c and d) The levels of p-MAPK6, MAPK6, p-MK5, MK5 and YAP in mouse renal tissue were evaluated by Western blot. **P < 0.01.

Finally, we further explored the mechanism by which MSC/miR-374a-5p-Exo regulated the development of renal fibrosis *in vivo*. As revealed in (Figures 6a-d), the expressions of p-MAPK6, p-MK5 and YAP were remarkably upregulated in UUO group, whereas these upregulations were notably reversed by MSC/miR-374a-5p-Exo treatment. All in all, MSC/miR-374a-5p-Exo could inhibit the progression of renal fibrosis *in vivo* by regulating MAPK6/MK5/YAP axis.

## Discussion

It has been reported that miR-374a-5p was under-expressed in renal venous blood samples from the patients with CKD [24]. We found that the expression of miR-374a-5p was downregulated by TGF-β1 in HK-2 cells, illustrating that miR-374a-5p may be crucial in kidney-related diseases.

So far, there’s a lot of research that demonstrates that exosome may serve as a carrier for the treatment of many diseases [23,42]. For example, exosome-mediated delivery of miR-125a-5p derived from BMSCs might serve as a new therapeutic strategy for the treatment of osteoarthritis (OA) [29]. In addition, exosomes are vital in kidney pathophysiology by facilitating cell-to-cell transport of miRNAs [43]. Meanwhile, exosomes from miR-let7c-modified MSCs relieves the progression of renal fibrosis [22]. In the present study, we found exosomes was able to transfer miR-374a-5p from MSCs to HK-2 cells and exert anti-fibrosis effects for the first time, indicating that exosomal miR-374a-5p was a hopeful therapeutic method for renal fibrosis.

It has been reported that MAPK6 play a vital role during the pathologic of kidney-related diseases [40]. For instant, high expression of MAPK6 in diabetic nephropathy could induce podocyte injury [40]. In this study, we found that TGF-β1 obviously promoted the level of p-MAPK6 in HK-2 cells. As we know, MK5 could be activated by MAPK6, and YAP could be activated by MK5 [44]. For example, Nawaito et al. showed that MK5 was a downstream protein of MAPK6 [44] and Seo indicated that YAP was a downstream protein of MK5 [45]. Current data had concluded that MSC/miR-374a-5p-Exo markedly inhibited TGF-β1-induced apoptosis of HK-2 cells and prevented the progression of renal fibrosis *in vivo* by regulating MAPK6/MK5/YAP axis, suggesting that MAPK6/MK5/YAP was vital in the pathogenesis of renal fibrosis.

Frankly speaking, there are some limitations needed to be improved in the coming study. For example, we only confirmed the relationship between miR-374a-5p and MAPK6 in the pathological process of renal fibrosis. Indeed, other potential targets such as WNT5A, BMP2 and WNT3 were found in the database, which were not verified yet. Moreover, the current studies have found that MAPK6/MK5/YAP axis was involved in the process of renal fibrosis inhibited by miR-374a-5p modified exosomes, and it is worth exploring whether other axes are also involved.

## Conclusion

In conclusion, exosomes from miR-374a-5p-modified MSCs prevented the progression of renal fibrosis by regulating MAPK6/MK5/YAP axis. Thus, our study might provide a new therapeutic way for renal fibrosis.

## Supplementary Material

Supplemental Material

## Data Availability

The datasets used and/or analyzed are available from the corresponding author on reasonable request.
