# Supplementary figures and images for: Exosomes from miR-374a-5p-modified mesenchymal stem cells inhibit the progression of renal fibrosis by regulating MAPK6/MK5/YAP axis
Source: Bioengineered. 2022 Feb 9;13(2):4517–27. doi: 10.1080/21655979.2022.2033465 (PMC8973867; doi:10.1080/21655979.2022.2033465)

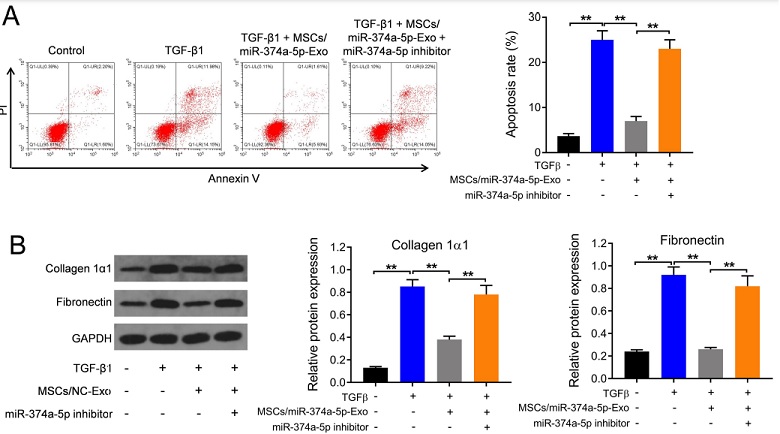

Supplement: Supplemental Material [file KBIE_A_2033465_SM0057.jpg]
